# Supplementary material for: Optical vectorial-mode parity Hall effect: a case study with cylindrical vector beams
Source: Nat Commun. 2024 May 13;15:4022. doi: 10.1038/s41467-024-48187-3 (PMC11091081; doi:10.1038/s41467-024-48187-3)
Supplement: Supplementary file 1 — Supplementary Information [file 41467_2024_48187_MOESM1_ESM.pdf]

Supplementary information

Optical vectorial-mode parity Hall effect：a case study with cylindrical vector beams

Changyu Zhou[1,#], *Weili Liang*[1,#], *Zhenwei Xie*[1,#,*], *Jia Ma*[1], *Hui Yang*[2], *Xing Yang*[2], *Yueqiang Hu*[2,3], *Huigao Duan*[2,3,*], *Xiaocong Yuan*[1,4,*]

[1]Nanophotonics Research Center, Institute of Microscale Optoelectronics & State Key Laboratory of Radio Frequency Heterogeneous Integration, Shenzhen University, Shenzhen 518060, China.
[2]National Research Center for High-Efficiency Grinding, College of Mechanical and Vehicle Engineering, Hunan University, Changsha 410082, China.
[3]Greater Bay Area Institute for Innovation, Hunan University, Guangzhou 511300, Guangdong Province, China.
[4]Research Centre for Frontier Fundamental Studies, Zhejiang Lab, Hangzhou 311100, China.
[#]These authors contributed equally to this work.
[*]Correspondence: ayst31415926@szu.edu.cn; duanhg@hnu.edu.cn; xcyuan@szu.edu.cn

CONTENTS

S1. Parity for the vectorial optical field 2

S2. Metasurface design for the realization of vectorial-mode parity Hall effect 4

S3. Demultiplexing of parity and topological charge in multiple vector modes 7

S4. Parity and topological-charge demultiplexed CVB encoded meta holography 9

S1. Parity for the vectorial optical field

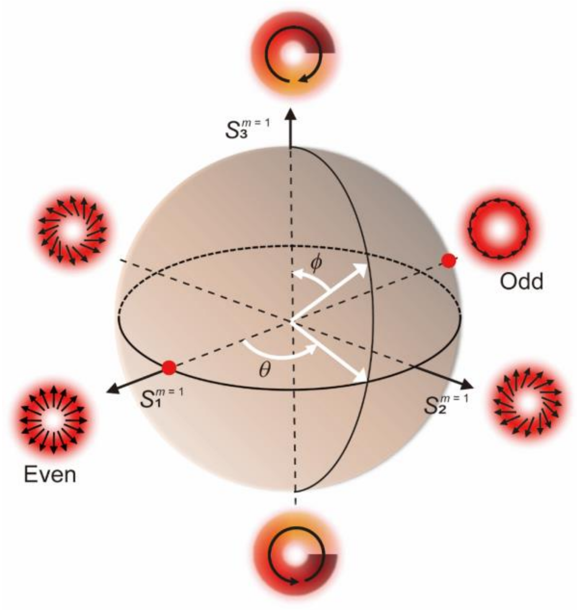

Figure S1. VOFs with the representation in 1st-order Poincaré sphere. There are two points on the sphere that corresponds to the even and odd mode.

Taking into account the general expression of a vectorial optical field (VOF), which is obtained by superimposing states on the hybrid-order Poincaré sphere, given by [1]

$$\mathbf{\Psi}_{LG_p^{m_1,m_2}}(\mathbf{r}) = \cos\left(\frac{\phi}{2}\right)\mathrm{LG}_p^{m_1}e^{i\theta/2}\hat{\mathbf{e}}_R + \sin\left(\frac{\phi}{2}\right)\mathrm{LG}_p^{m_2}e^{-i\theta/2}\hat{\mathbf{e}}_L, \tag{S1}$$

where ϕ and θ are orientation angle and ellipticity angle on the sphere, respectively. $\hat{\mathbf{e}}_R$ ($\hat{\mathbf{e}}_L$) represents the unit vector of the right (left) circular polarization, m_1 (m_2) is azimuthal index (topological charge) and p is radial index of the Laguerre-Gaussian (LG) modes, defined by

$$\begin{aligned}\mathrm{LG}_p^m(r,\varphi,z) = &\sqrt{\frac{2p!}{\pi(|m|+p)!\omega^2(z)}}\left(\frac{\sqrt{2}r}{\omega(z)}\right)^{|m|} \\ &\times \mathrm{L}_p^{|m|}\left(\frac{2r^2}{\omega^2(z)}\right)\exp[i(2p+|m|+1)\zeta(z)] \\ &\times \exp\left(\frac{-r^2}{\omega^2(z)}\right)\exp\left(\frac{-ikr^2}{2R(z)}\right)\exp(im\varphi),\end{aligned} \tag{S2}$$

where $\mathrm{L}^m{}_p$ represents the generalized Laguerre polynomial, $R(z)$, $\omega(z)$ and $\zeta(z)$ are the radius of curvature, Gaussian beam radius and Gouy phase, respectively. (r, φ, z) are the cylindrical-coordinate parameters.

The parity of a VOF is determined by the polarization distributions in the transverse plane of the beam, and can be precisely defined through

$$\hat{P}\boldsymbol{\psi}(\hat{P}\mathbf{r}) = P\boldsymbol{\psi}(\mathbf{r}), \tag{S3}$$

where $\hat{P}$ is the parity operator and $P = \pm 1$ is the corresponding eigenvalue, $\boldsymbol{\psi}(\mathbf{r})$ is the target state of polarization (SOP) of a VOF. Without loss of generality, we focus on the azimuthal dependent VOFs in Eq. (S1). Therefore, Eq. (1) can be simplified in the $\hat{\mathbf{e}}_R$-$\hat{\mathbf{e}}_L$ basis by

$$\boldsymbol{\Psi}(\mathbf{r}) = \exp(im_+\varphi)\boldsymbol{\psi}(\mathbf{r}) = \exp(im_+\varphi)\begin{pmatrix} \cos\left(\dfrac{\phi}{2}\right)e^{i\theta/2}\exp(im_-\varphi) \\ \sin\left(\dfrac{\phi}{2}\right)e^{-i\theta/2}\exp(-im_-\varphi) \end{pmatrix}, \tag{S4}$$

where $m_\pm = (m_1 \pm m_2)/2$. In the situation of $m_1 = -m_2 = 1$, that is, Eq. (S4) can be denoted as the SOPs on the 1st-order Poincaré sphere, as shown in Fig. S1. Additionally, for a pair of SOPs with $\theta = 0$ and π, while ϕ is fixed at $\pi/2$, namely the states given by

$$\begin{aligned} \boldsymbol{\Psi}_1(\mathbf{r}) &= \frac{1}{\sqrt{2}}\exp(im_+\varphi)\begin{pmatrix} \exp(im_-\varphi) \\ \exp(-im_-\varphi) \end{pmatrix}, \\ \boldsymbol{\Psi}_2(\mathbf{r}) &= \frac{1}{\sqrt{2}}\exp(im_+\varphi)\begin{pmatrix} i\exp(im_-\varphi) \\ -i\exp(-im_-\varphi) \end{pmatrix}, \end{aligned} \tag{S5}$$

such two SOPs corresponds to the vector vortex modes. The corresponding eigenvalue for the SOPs of $\boldsymbol{\Psi}_1(\mathbf{r})$ and $\boldsymbol{\Psi}_2(\mathbf{r})$, according to Eq. S3, are $P = 1$ and $P = -1$, respectively. Here $\hat{P}$ denotes the x-axis mirror operator σ_z (the z component of Pauli matrices) in the $\hat{\mathbf{e}}_x$-$\hat{\mathbf{e}}_y$ basis (or σ_x in the $\hat{\mathbf{e}}_R$-$\hat{\mathbf{e}}_L$ basis). In the situation of $m_1 = -m_2 = 1$, the radially polarized CVB is of even parity ($P = 1$), and the azimuthally polarized CVB is of odd parity ($P = -1$).

S2. Metasurface design for the realization of vectorial-mode parity Hall effect

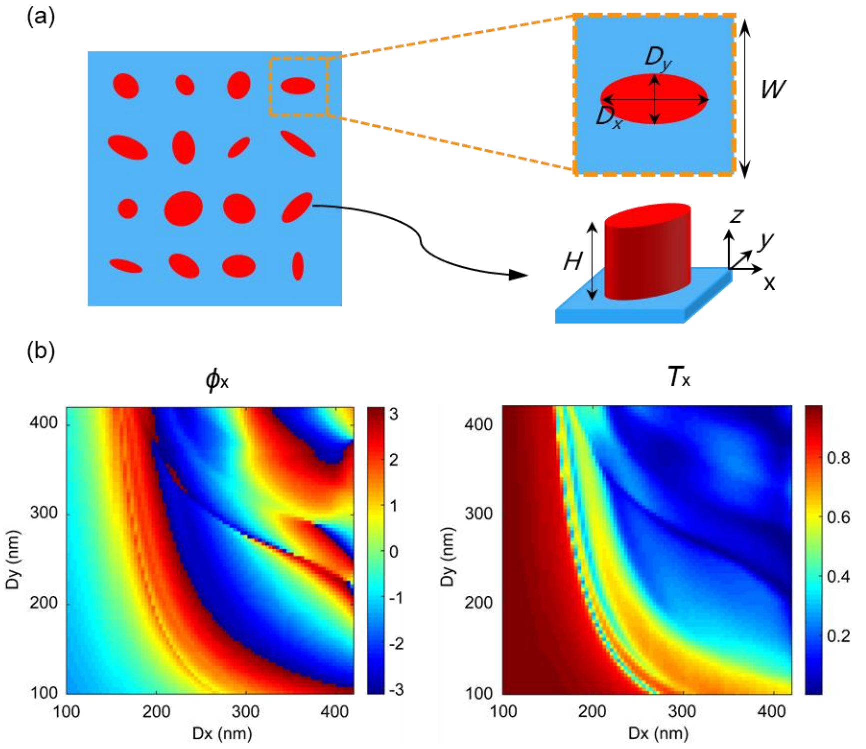

Figure S2. Simulation parameters. (**a**) A schematic diagram illustrating the design of the form-birefringent metasurface and its basic unit cell. All the nanopillars in the metasurface have a uniform height of $H = 1000$ nm, while the period of a single unit cell is set at $W = 500$ nm. However, the lengths D_x and D_y for two axes of the elliptical nanopillars vary. (**b**) Results obtained from finite-difference time-domain (FDTD) numerical simulations demonstrate the modulation of the phase ϕ_x and the transmittance T_x by altering D_x and D_y, ranging from 100 nm to 420 nm

The metasurface employed in this study comprises an array of periodically arranged sub-wavelength elliptical nanopillars made of Titanium Dioxide (TiO_2), positioned on a silica substrate. Each nanopillar acts as a weak coupling low-quality-factor resonator, exhibiting a strong electric field response. This enables independent phase and amplitude modulation along its two axes when illuminated by incident light, resulting in a form-induced birefringence effect. All nanopillars within the metasurface share a uniform height of $H = 1000$ nm, while the period of a single unit cell is $W = 500$ nm, as depicted in Fig. S2a. Fig. S2b showcases simulation results obtained through finite-difference time-domain (FDTD) analysis, demonstrating the phase response ϕ_x and transmittance T_x when subjected to an incident x-polarized beam within the simulation. The scanning parameters include the two main axis variables D_x and D_y of the elliptical nanopillars, with the scanning range spanning from 100 nm to 420 nm. It is worth noting that due to the symmetrical nature of the structure, the phase response ϕ_y and the transmittance T_y are the transpose equivalents of ϕ_x and T_x, respectively. From the results, it is evident that the phase response covers the entire range of 2π. The detailed parameters D_x and D_y that correspond to the desired modulated phase (ϕ_x and ϕ_y) are shown in Table S1 and S2, respectively.

Table S1. Parameters of D_x (nm)

ϕ_y \ ϕ_x	-3π/4	-π/2	-π/4	0	π/4	π/2	3π/4	π
-3π/4	400	208	240	280	348	420	280	112
-π/2	156	188	220	248	300	384	420	100
-π/4	148	180	204	232	268	332	420	100
0	140	172	196	220	256	308	408	420
π/4	132	160	188	208	240	288	372	420
π/2	128	148	176	196	224	264	340	420
3π/4	420	148	164	180	204	240	304	372
π	212	272	276	180	200	228	284	136

Table S2. Parameters of D_y (nm)

ϕ_y \ ϕ_x	-3π/4	-π/2	-π/4	0	π/4	π/2	3π/4	π
-3π/4	404	156	148	140	132	128	420	212
-π/2	208	192	180	172	160	148	148	272
-π/4	240	220	208	196	188	176	164	276
0	280	248	232	220	208	196	180	180
π/4	348	300	268	256	240	224	204	200
π/2	420	384	332	308	288	264	240	228
3π/4	280	420	420	408	372	340	304	284
π	112	100	100	420	420	420	372	136

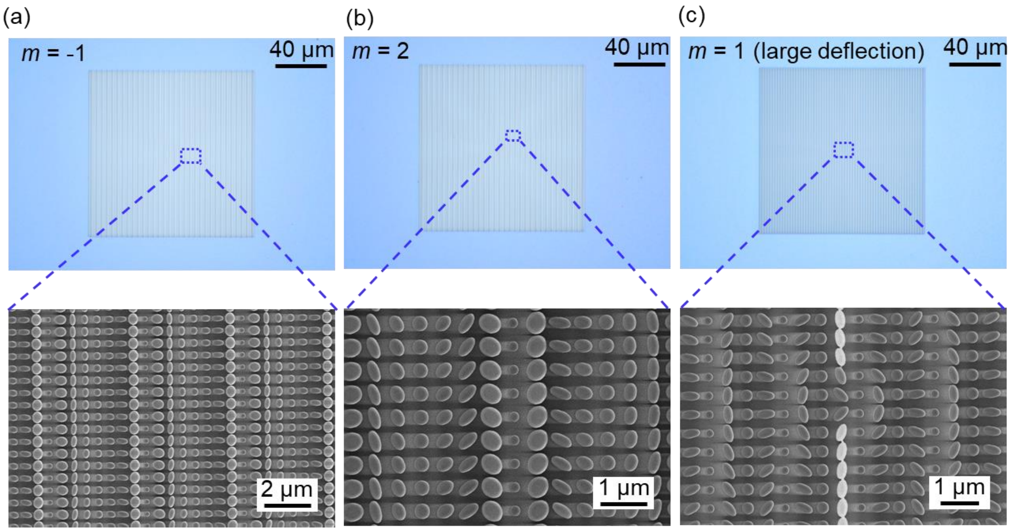

Figure S3. Microscopy and SEM images of the fabricated samples. (**a-c**) Microscopy and SEM images for the fabricated metasurfaces, with the functions of separating the topological charges (a) m = -1, (b) m = 2, (c) m = 1 (large deflected angle) of the CVBs.

To validate the effectiveness of the approach described in the main text, we have designed the metasurfaces that are capable of achieving parity splitting for ±1st- and 2nd-order cylindrical vector beams (CVBs). To achieve the desired phase modulation, careful selection of parameters (D_x and D_y) for the elliptical nanopillars was conducted based on the results

shown in Fig. S2b. The scanning electron microscope (SEM) of the fabricated samples are shown in Fig. S3. Fig. S4 presents the results obtained from finite-difference time-domain (FDTD) simulations and experimental tests conducted on the far-field distributions of the CVBs passing through the metasurfaces (also depicted in Fig. 3 of the main text). These results clearly demonstrate the parities of the vector mode of the CVBs are effectively separated in the desired directions, as confirmed by both simulation and experimental observations. Additionally, the deflection angle of the beam can be adjusted flexibly. Furthermore, we calculated the separated efficiencies for the even and odd CVBs by integrating the intensity distributions using the experimental findings presented in Fig. S4b. The calculated results are summarized in Table S3.

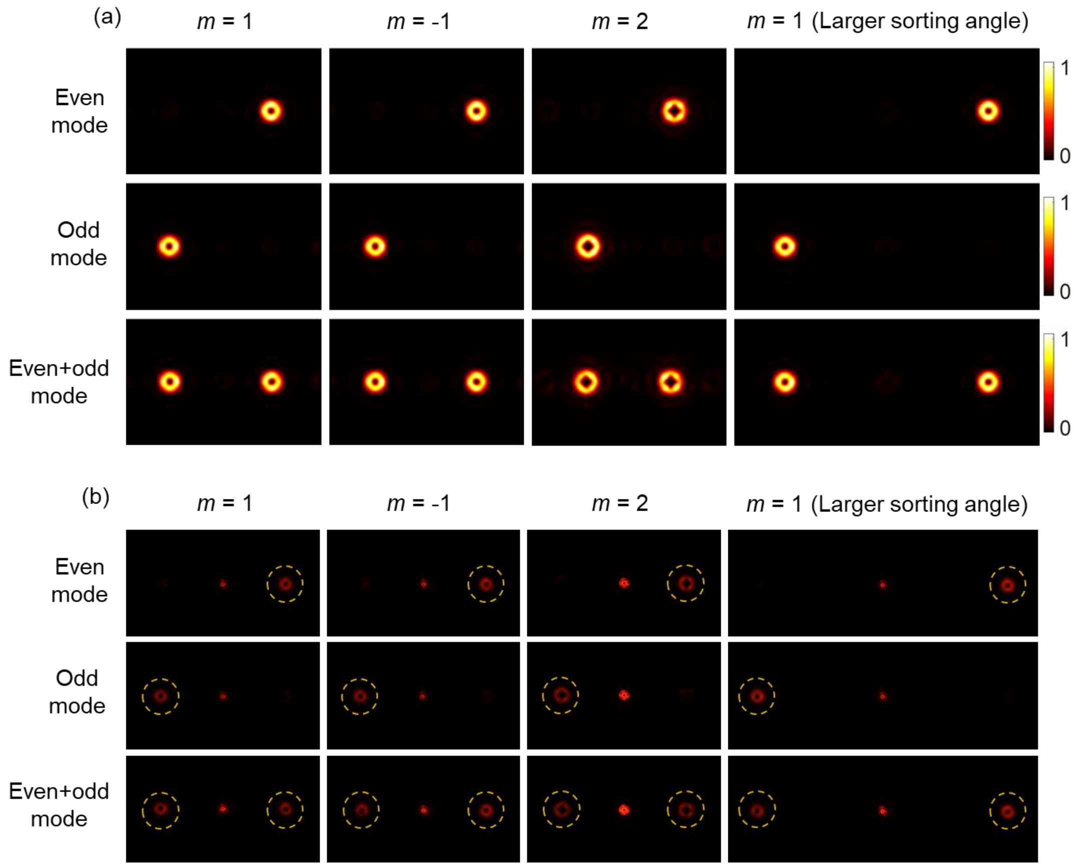

Figure S4. Verification of vectorial-mode parity Hall effect. (**a**) FDTD simulated far field results and (**b**) experimentally test results for the separation of even/odd CVB with topological charge $m = 1, -1$ and 2.

Table S3. Calculated efficiency for m_{th}-order CVB

Input mode	Output mode	Calculated efficiencies (%)			
		$m = 1$	$m = -1$	$m = 2$	$m = 1$(large deflection)
even	even	61.50%	60.63%	44.95%	67.30%
even	odd	7.34%	8.29%	4.21%	2.52%
odd	odd	59.03%	59.60%	39.07%	63.69%
odd	even	8.97%	9.28%	6.69%	2.82%

S3. Demultiplexing of parity and topological charge in multiple vector modes

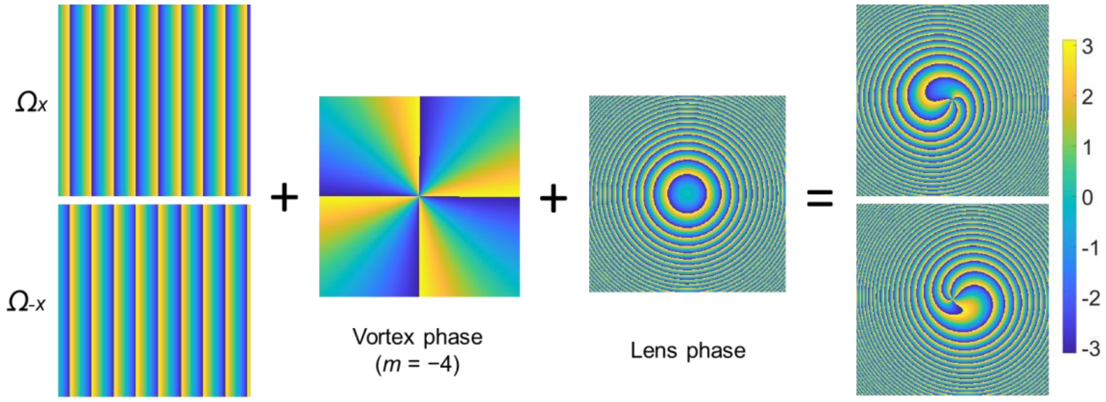

Figure S5. Schematic for converting separated CVB to the focused Gaussian beam.

The separated even and odd CVBs achieved by the metasurface still exhibit a hollow distribution and have limited coupling efficiency when coupled to the widely used single-mode fibers, especially for higher-order CVBs. In order to address this issue, we have introduced additional phase terms, namely the inverse-order vortex phase and the lens phase, to the metasurface. These phases, along with the separation phase distributions of Ω_x and Ω_{-x}, enable the conversion of the CVBs into the focused Gaussian beams. The diameter of the resulting focused beam can be adjusted to match the requirements of the coupled single-mode fiber. Figure S5 illustrates the schematic of this approach, using the 4th-order CVB as an example. The added vortex phase is set to $m = -4$, and a lens with a focal length of 1 μm is incorporated into the system.

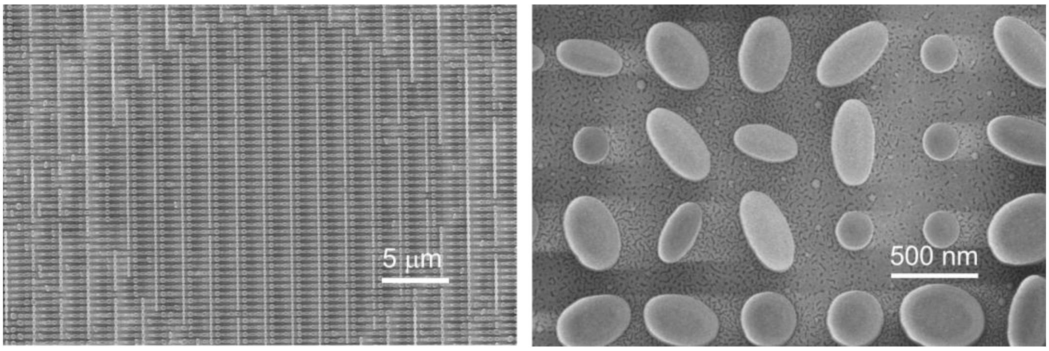

Figure S6. SEM images of the fabricated metasurface for CVB demultiplexing.

We have successfully designed a metasurface capable of demultiplexing CVBs of up to 14 channels, where the topological charge- and parity-demultiplexed CVBs are evenly distributed on a circular pattern. The scanning electron microscope (SEM) of the fabricated samples are shown in Fig. S6. The diameter of the designed metasurface is 250 μm, and the simulated results are depicted in Fig. S7. As observed, the desired CVB produces focused Gaussian spots at their respective positions on the image plane. It is worth noting that there is a relatively larger overlapping area among the high-order CVBs compared to the low-order ones, leading to unavoidable cross talk between these channels (refer to Fig. 4a in the main text). However, the additional inverse-vortex phase and the lens phase significantly improves the efficiency of the

CVBs coupled to the single-mode fiber by generating the focused Gaussian spots. Conversely, the coupling efficiencies for the undesired CVBs are suppressed due to mode mismatching, as demonstrated in Figure S7b. A Gaussian spot is precisely generated at the desired position, while the light spots with side petals are effectively prevented from coupling into the corresponding single-mode fiber. This achievement enables the demultiplexing of CVBs while eliminating cross talk between different channels.

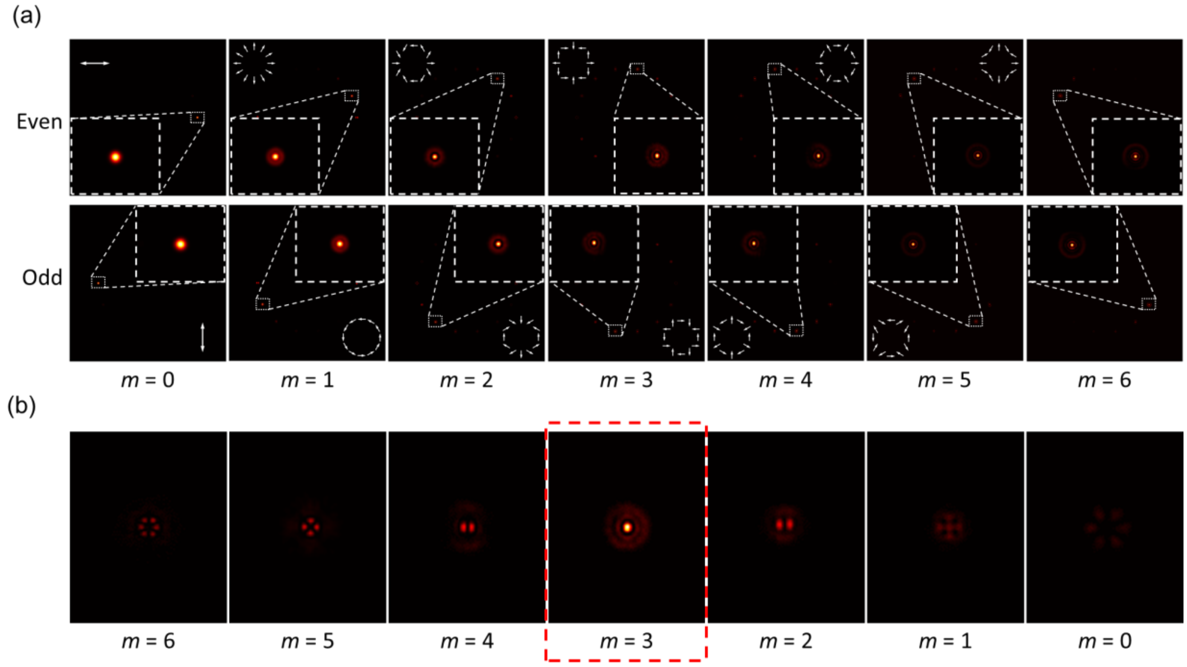

Figure S7. Demultiplexing of Topological Charge and Parity in CVBs. (**a**) Theoretically calculated results for demonstrating the demultiplexing of CVBs up to 14 channels, with the demultiplexed topological charge ranging from $m = 0$ to $m = 6$. The white-arrows pattern in each subgraph indicates the polarization distributions of the corresponding CVB. (**b**) Calculated results for showcasing the input CVB with a topological charge of $m = 3$, where the desired outcome is a focused Gaussian spot, while the other channels exhibit different distributions.

S4. Parity and topological-charge demultiplexed CVB encoded meta holography

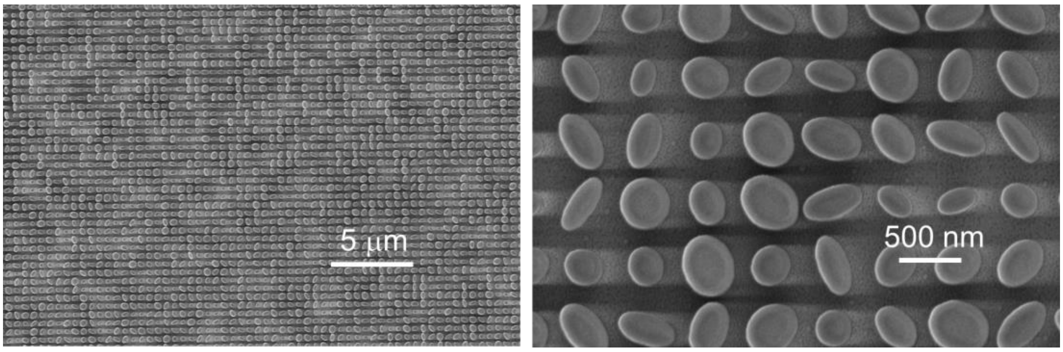

Figure S8. SEM images of the fabricated metasurface for CVB encoded meta holography.

Taking advantage of the additional parity dimension present in CVBs, we have successfully achieved the encoding of holographic plays using 8-channel CVBs through a super unit cell design, which consists of four staggered unit cells within a single metasurface (refer to Fig. 5b in the main text). The scanning electron microscope (SEM) of the fabricated samples are shown in Fig. S8. The simulated and experimentally tested results are illustrated in Fig. S9 and Fig. 5, respectively. To mitigate the cross talk between different channels, we incorporated inverse-vortex phases of varying orders into the metasurface, as described in section S3. As a result, only when the corresponding-order CVB is incident, can the formation of Gaussian light spots occur. This meticulous design ensures that the desired Gaussian light spots are specifically generated for each channel while minimizing interference from other channels.

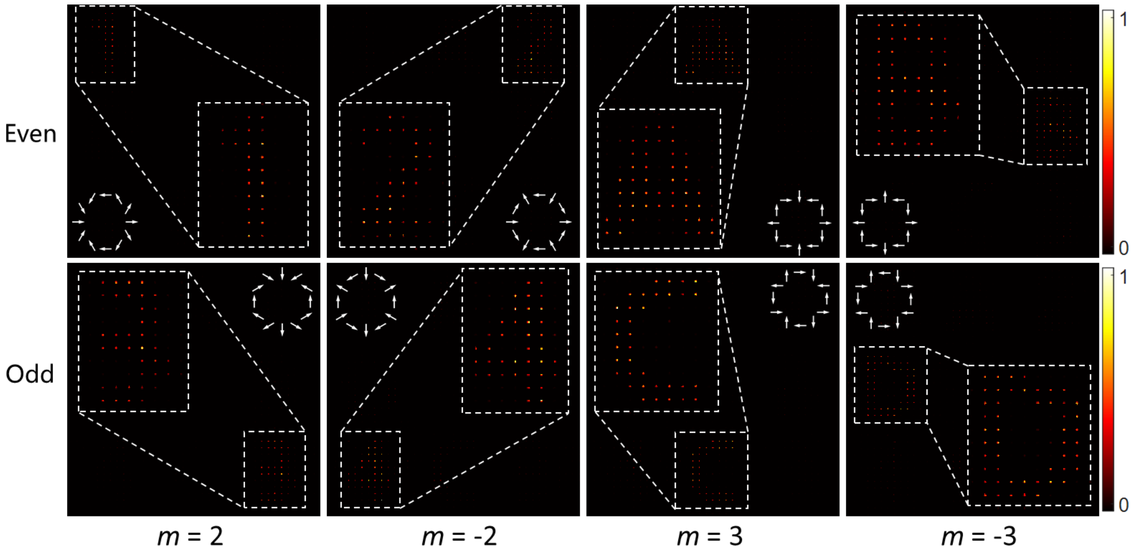

Figure S9. Theoretically calculated results for the CVB demultiplex holography with topological charge $m = \pm 2, \pm 3$ and the even/odd parity.

References

[1] C. Rosales-Guzmán, B. Ndagano, A. Forbes. A review of complex vector light fields and their applications. *Journal of Optics*, **20**, 123001 (2018).
